# The mTOR/Akt pathway is involved in regulating astrocyte growth and GLT-1 expression during cerebral ischemia-reperfusion

**DOI:** 10.1371/journal.pone.0351107

**Published:** 2026-06-12

**Authors:** Qiuling Li, Yiwen Yu, Huan Deng, Biao Li, Hao Zhao, Dong Lei, Mi Li, Shansha Xie, Jingmei Yu, Yuping Zhao, Shubin Yin, YiFei Ji

**Affiliations:** 1 Department of Neurology, Second Clinical College, North Sichuan Medical College, Nanchong, China; 2 Department of Neurology, Nanning First People’s Hospital, Nanning, China; 3 Department of Neurology, Yilong County People’s Hospital, Nanchong, China; 4 Department of Neurology, Ya ‘an People’s Hospital, Ya ‘an, China; Southern Illinois University Carbondale, UNITED STATES MINOR OUTLYING ISLANDS

## Abstract

Stroke is one of the most prevalent causes of death and disability worldwide and places a heavy economic burden on families and society. High glutamate accumulation and subsequent excitotoxicity after ischemia and hypoxia are important pathogenetic mechanisms of brain injury after ischemic stroke. Glutamate transporter 1 (GLT-1) on astrocyte membranes is responsible for 90% of glutamate clearance. The involvement of the mTOR/Akt pathway in the upregulation of GLT-1 expression in astrocytes under oxygen-glucose deprivation and reoxygenation conditions has been demonstrated. Nevertheless, it is still unclear whether there is a negative feedback pathway from mTOR to Akt during cerebral ischemia/reperfusion (I/R). It has also not been elucidated whether the mTOR/Akt pathway is involved in the expression of astrocyte GLT-1 in cerebral I/R injury. In this study, we established a middle cerebral artery occlusion-reperfusion rat model to investigate the interactions and mechanisms of the mTOR/Akt cascade with GLT-1 under cerebral I/R conditions. These results provide evidence that brain I/R injury activates the mTOR/Akt pathway in the ischemic penumbra, increases astrocyte activation, and downregulates the expression of GLT-1. Inhibition of the mTOR pathway reversed GLT-1 downregulation and inhibited astrocyte activation by blocking the mTOR pathway, thereby attenuating neurological dysfunction, inflammatory response, and apoptosis caused by brain I/R injury. In contrast, inhibiting the Akt pathway did not provide neuroprotection, with no significant decrease in the number of astrocytes, inflammatory response, or apoptosis in the model group. Additionally, the inhibition downregulated GLT-1 expression and promoted the lengthening and thickening of astrocyte processes in cerebral ischemic rats. Thus, the mTOR/Akt cascade may be involved in regulating astrocyte growth and GLT-1 expression during brain I/R injury. Furthermore, inhibiting the mTOR pathway may mitigate apoptosis and the release of inflammatory factors, thereby fostering neuronal survival and safeguarding the central nervous system.

## Introduction

Stroke is the second leading cause of death and disability among adults globally [1]. It poses a serious threat to human life and health, with 6.55 million stroke-related deaths predicted in 2019 [2]. Stroke is clinically classified into two basic categories: hemorrhagic and ischemic [3]. Ischemic stroke (IS) accounts for 87% of all stroke cases in the United States [1]. It is a clinical syndrome characterized by cerebral artery occlusion, which disrupts the blood supply to the brain, causing ischemia and hypoxic necrosis of local brain tissue. This, in turn, leads to various neurological deficits [4]. The most common sequelae include motor, speech, and vision deficits, and approximately half of those who survive stroke have residual cognitive or physical impairments, all of which severely affect their quality of life [5,6]. At present, mechanical thrombectomy, recombinant tissue plasminogen activator, and urokinase thrombolysis are the current specific treatments for patients with acute IS; however, the narrow time window for treatment and the danger of bleeding limit their benefits to only 5% of patients with stroke [7–9]. Revascularization may save vulnerable neurons over time; nonetheless, the resulting ischemia-reperfusion (I/R) injury may further destroy the brain tissue [10]. Recently, neurons have been targeted in stroke care strategies, but little clinical efficacy has been observed [11,12]. However, the brain is also populated by other cell types, including astrocytes, which outnumber neurons by more than five to one and are responsible for supplying neurons with energy metabolites, regulating ion homeostasis, and participating in synaptic function, which may account for treatment failure using target neurons alone [13].

Astrocytes make up approximately 50% of the volume of the human brain and are the most abundant glial cell subtype in the mammalian central nervous system (CNS) and the most abundant cell type in the CNS [14]. Their key functions include neuronal support, regulation of cerebral blood flow (CBF) and intracellular and extracellular ion concentrations, promotion of neurotransmitter synthesis and clearance, glucose supply, antioxidant defense, and regulation of synaptic activity [15]. Notably, astrocytes may play a dual role in brain I/R injury. These cells are activated following cerebral ischemia [1]. During brain injury, astrocytes conserve glycogen and energy, promote the expression and release of neurotrophic factors (nerve growth factor, brain-derived growth factor, and vascular endothelial growth factor), and reabsorb excess glutamate (Glu) in the synaptic cleft to protect ischemic neurons [16]. Nevertheless, activated astrocytes can also secrete pro-inflammatory factors such as interleukin (IL)-1β and tumor necrosis factor (TNF)-α to inflict damage, as well as form neuroglial scars to impede axonal regeneration [17]. Short-term administration of the mammalian target of rapamycin (mTOR) inhibitor rapamycin (RAPA) effectively curtails astrocyte proliferation and inflammatory responses following CNS injury, consequently ameliorating neuronal death and enhancing motor function [18]. Therefore, the role of astrocytes in brain I/R injury cannot be ignored. Given the beneficial and detrimental effects of these cells in stroke, enhancing their favorable effects may be a promising approach for treating related diseases.

The pathophysiological mechanisms underlying brain I/R injury include Glu excitotoxicity, Ca^2+^ overload, oxidative stress, autophagy, inflammation, and apoptosis [19]. Recently, the important role of excitatory amino acid toxicity in various neurological disorders has received increasing attention, including stroke, amyotrophic lateral sclerosis, Alzheimer’s disease, Parkinson’s disease, manganese toxicity, schizophrenia, epilepsy, and autism [20–22]. Glu is the dominant excitatory neurotransmitter in the CNS; during cerebral ischemia, massive accumulation of Glu in the extracellular space overexcites its receptors on neurons and induces excess Ca^2+^ in neurons, causing excitotoxic neuronal death [23,24]. The excitatory amino acid transporter Glu transporter-1 (GLT-1) is crucial for the removal of excess Glu. GLT-1 is predominantly expressed in astrocytes and is responsible for approximately 90% of Glu removal from the synaptic gap [25]. Astrocytes remove most of the extracellular Glu via GLT-1. The Glu that enters astrocytes is metabolized by glutamine synthetase into glutamine, which is released into the extracellular space and taken up by presynaptic neurons to resynthesize Glu or gamma-aminobutyric acid, preventing excitotoxicity from excess Glu [24]. GLT-1 expression is depressed, and extracellular Glu reuptake is reduced after brain I/R injury [26]. Therefore, we postulate that the dysregulation of GLT-1 may contribute substantially to the excitotoxicity and pathogenesis of IS.

However, to date, the mechanisms that modulate the expression of GLT-1 in cerebral ischemic-hypoxic injury have been poorly understood, and there are no approved pharmacological treatments aimed at upregulating GLT-1 expression. Previous studies have revealed several regulatory mechanisms of GLT-1 expression. Sulbactam exerts neuroprotective effects in hippocampal neurons by upregulating GLT-1 expression in ischemic rats by activating p38 mitogen-activated protein kinases [27]. The estrogen receptor modulators 17β-estradiol and tamoxifen promote upregulation of GLT-1 expression in astrocytes via epidermal growth factor (TGF-α) signaling [28]. Karki et al. previously reported that mitogen-activated protein kinase/extracellular signal-regulated kinase and phosphatidylinositol 3-kinase/Protein Kinase B (Akt) promote NF-κB activation [29]. In our preceding report, we found that the mTOR-Akt-nuclear factor-кB signaling cascade mediates the upregulation of GLT-1 expression under oxygen-glucose deprivation (OGD) conditions [30]. We verified through our *in vitro* experiments the involvement of the mTOR/Akt pathway in enhancing GLT-1 in astrocytes during oxygen-glucose deprivation and reoxygenation (OGD/R) [31]. Nevertheless, the underlying mechanisms of the mTOR/Akt pathway and GLT-1 in brain I/R conditions are not yet fully understood.

Accordingly, we used a middle cerebral artery occlusion and reperfusion (MCAO/R) model to investigate the role of the mTOR/Akt cascade in the ischemic penumbra in regulating the expression of GLT-1 and astrocyte proliferation after I/R injury in the rat brain. We also examined the influence of RAPA on GLT-1 expression in the rat brain following I/R injury. Our research provides novel insights into the role of the mTOR-Akt cascade in the central nervous system, which may lead to innovative therapeutic approaches for stroke.

## Materials and methods

### Antibodies and reagents

Antibodies against phospho-S6-Ser240/244 (pS6) and phospho-Akt-Ser473 (pAkt) were purchased from Cell Signaling (USA). EAAT2 (GLT-1), glial fibrillary acidic protein (GFAP), neuron-specific nuclear protein (NeuN), β-actin, phospho-mTOR (Ser2448), mTOR, Akt, S6, Bcl-2, Bax primary antibodies, Horseradish Peroxidase (HRP)-Conjugated Goat anti-rabbit, HRP-Conjugated Goat anti-mouse, iFluor^TM^ 488 Conjugated Goat anti-mouse, and iFluor^TM^ 594 Conjugated Goat anti-rabbit secondary antibodies were purchased from Huabio (China). β-Tublin was purchased from Zenbio (China). RAPA, tricitribine (TCBN), polyethylene glycol (PEG) 300, and tween 80 were purchased from MCE (USA). 2, 3, 5-triphenyltetrazolium chloride (TTC) was purchased from Solarbio (China). Enzyme-linked immunosorbent assay ELISA kits were purchased from FineTest (China). The TUNEL apoptosis kit was purchased from Elabscience (Beijing, China).

### Experimental animals and ethics statement

Male Sprague Dawley (SD) rats (230–260 g, 6–8 weeks) were provided by the Animal Center of North Sichuan Medical College. Male rats were selected for all experiments to control the potential effects of cyclical changes in estrogen on stroke [32]. Animals were transferred to cages containing sterile wood shavings as bedding material and allowed to rest for a week in controlled environmental conditions (22–24°C, 12/12 h light/dark cycle, 55% ± 10% relative humidity) to adapt to the new environment. The rats had free access to food and water. The experimental procedures began after a 7-day adaptive feeding period. The SD rats were randomly divided into seven groups: Sham, Vehicle + Sham, RAPA + Sham, TCBN + Sham, Vehicle + MCAO/R, RAPA + MCAO/R, and TCBN + MCAO/R. All operators were blind to the groups. All animals were subjected to neurobehavioral tests. The health and behavior of the animals were monitored daily. Three animals from each group were used for western blot analyses, immunofluorescence, and TTC staining. This study was performed following the Animal Research Reporting of *In Vivo* Experiments (ARRIVE) guidelines. The experimental protocols were approved by the Animal Welfare and Ethical Review Committee of North Sichuan Medical College [Permit number: NSMC-2022031]. All surgeries were performed under sodium pentobarbital anesthesia, and efforts were made to minimize suffering.

### Drug administration

**Fig 1:**
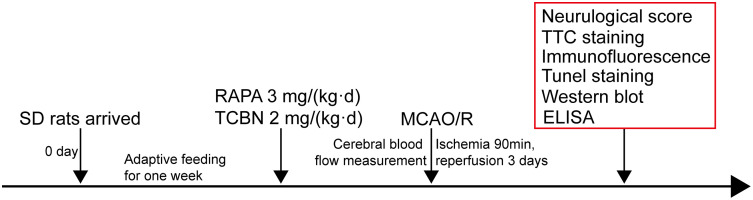
Flow chart of the study design. Rapamycin and triciribine are mTOR and Akt inhibitors, respectively. RAPA: rapamycin; TCBN: triciribine; i.p: intraperitoneal; MCAO/R: middle cerebral artery occlusion and reperfusion; TTC: 2, 3, 5-triphenyltetrazolium chloride; ELISA: enzyme-linked immunosorbent assay.

RAPA and TCBN stock solutions were prepared by dissolving 100 mg of RAPA (200 mg/ml, MCE) and 50 mg of TCBN (100 mg/ml, MCE) in 0.5 ml dimethyl sulfoxide (DMSO, MP Biomedicals), which was stored at −80°C for future use. The working solution was prepared by mixing the stock solution with 3% Tween 80 and 15% PEG 300 to a final concentration of 1% DMSO immediately before intraperitoneal (i.p.) injection. Seven days before ischemia induction, the RAPA+ Sham and RAPA+ MCAO/R groups were injected daily with RAPA (3 mg/kg/day) [33], and the TCBN+ Sham and TCBN+ MCAO/R groups were injected daily with TCBN (2 mg/kg/day) until the animals were sacrificed [34]. The vehicle + sham and vehicle + MCAO/R groups were injected with equal amounts of vehicle (3% Tween 80, 15% PEG 300, and 1% DMSO). A flow diagram of the animal protocol is shown in Fig 1.

### MCAO/R model establishment

**Fig 2:**
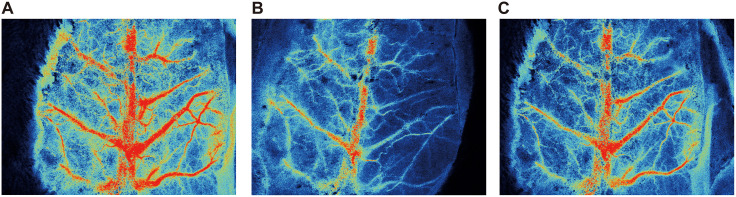
Detection of cerebral blood flow by laser speckle blood flow imager. **(A)** Normal cerebral cortical blood flow distribution of rats. **(B)** After occlusion of the right middle cerebral artery, cerebral blood flow in rats was measured. The results showed that the blood flow in the right hemisphere was blocked. **(C)** Cerebral blood flow in rats after removal of the wire embolus (following reperfusion): Right cerebral blood flow was almost fully restored.

Rats were anesthetized intraperitoneally using 0.1% sodium pentobarbital, and CBF was measured using a laser speckle blood flow imager (SIM Opto-Technology Co., Ltd., China) before and after modeling to verify whether the model was successfully constructed. The MCAO/R model was built using the Zea Longa method [35]. The rats were restrained in the supine position, and a thermostatic heating pad was used to maintain the rectal temperature at 37 ± 0.5°C during the operation. The right common, external, and internal carotid artery (ICA) were entirely exposed. an incision was made using ophthalmic scissors in the external carotid artery near the bifurcation. Subsequently, a silicone rubber-coated nylon monofilament with a tip diameter of 0.35 ± 0.02 mm (RWD, China) was inserted into the ICA to approximately 18–20 mm depth. A decrease in baseline CBF to less than 30% was considered a successful MCAO, as shown by laser scattering [36] (Fig 2). Reperfusion was achieved by removing the wire 90 min after ischemia. In sham rats, monofilament nylon wires were placed in the external carotid artery rather than in the ICA [37].

### Neurological deficit evaluation, inclusion, and exclusion criteria

Neurological deficits were graded using the Longa scale (Table 1) after the rats were fully awake and reperfused for 3 days [38]. Only rats with scores of 1, 2, and 3 were included in the follow-up experiments. MCAO/R was deemed effective only if CBF decreased to < 30% and returned to > 70% of baseline [36]. Animals that did not meet this criterion and scored 0 or 4 were discarded and supplied in time. None of the animals that died had been found before meeting euthanasia criteria.

**Table 1:** Zea-Longa score.

Score	Method
0	No neurological deficits were detected.
1	The front left paw is not fully extended.
2	Turn around to the left.
3	Rotate to the left and tilt to the side.
4	Inability to walk spontaneously or stroke-related mortality

### Infarct volume measurement

The rats that were reperfused for three days underwent deep anesthesia through intraperitoneal injection of 0.1% sodium pentobarbital. Subsequently, the brains were rapidly removed. The brain tissues were frozen at −80°C for 10 min, and 2-mm coronal slices were uniformly cut. The slices were subsequently incubated with 1% TTC (Solarbio) at 37°C for 30 min under light protection. Following this, the brain slices were soaked in 4% paraformaldehyde (PFA) and fixed at room temperature for 24 h. Next, the slices were photographed, and the infarcted area was quantified by two blinded researchers using ImageJ software. Infarct volume (%) = (contralateral hemisphere volume – ipsilateral hemisphere non-infarct volume)/contralateral hemisphere volume × 100%.

### Preparation of paraffin sections and tissue sample collection

Three days post-reperfusion, rats were intraperitoneally injected with 0.1% pentobarbital sodium for deep anesthesia. Subsequently, the rats were subjected to heart perfusion with 0.01M phosphate-buffered saline (PBS) followed by 4% PFA. Brain slices were harvested from the optic chiasm, 2 mm posterior to the infundibular stalk, and were immersed in 4% PFA and fixed at room temperature for 48 h. The brain slices were initially dehydrated by immersion in a low-to-high gradient of alcohol, then cleared using xylene, and finally ﬁlled with liquid paraffin for paraffin embedding. Paraffin sections of 5 μm thickness were prepared along the coronal plane of the brain and subsequently stored in a refrigerator at 20°C. To identify the protein expression of GLT-1 and the mTOR/Akt pathway, cerebral cortex samples were collected and frozen to −80°C in EP tubes for subsequent storage.

### Immunofluorescence analysis

Sections were first deparaffinized, hydrated, permeabilized with 0.4% Triton X-100 for 25 min, and followed with 10% goat serum for 30 min. The sections were incubated overnight with a monoclonal anti-phospho-S6 primary antibody (dilution 1:200) at 4°C. Brain sections were incubated overnight at 4°C with the following primary antibodies: mouse anti-GFAP (1:200, Huabio) to label astrocytes and rabbit anti-neuron-specific nuclear protein (NeuN, 1:300, Huabio). On the second day, the sections were incubated with iFluor^TM^ 488-labeled goat anti-mouse (Huabio, 1:600) and iFluor^TM^ 594-labeled goat anti-rabbit secondary antibodies (Huabio, 1:800) for 30 min. Cell nuclei were stained with 4’,6-diamidino-2-phenylindole (DAPI) and sealed with an anti-fluorescence quencher. The positive cells in the ischemic penumbra were photographed and statistically analyzed using a fluorescence microscope (Olympus). Three rats with successful experiments were selected from each group to ensure the reliability of the results. Two discontinuous sections were obtained from each rat, and three non-overlapping visual fields in each section were captured for measurement. We imported the images merged with DAPI into ImageJ software and manually calculated the number of cells in each picture using the cell counting function. Cells with overlapping green or red fluorescence signals and DAPI signals were considered positive. The same principle was used for each image. Finally, all data were imported into the GraphPad Prism software for statistical analysis.

### Terminal Deoxynucleotidyl Transferase-mediated dUTP Nick-end Labeling (TUNEL) assay

Apoptosis in the ischemic penumbra of the cerebral tissue was detected using a TUNEL Apoptosis Detection Kit (Elabscience, China), following the manufacturer’s protocol. Briefly, the animals were anesthetized, and brain samples were removed and fixed in 4% PFA. Following this, the samples were embedded in paraffin and sectioned coronally at 5 μm thickness. After dewaxing and rehydrating, the sections were incubated with proteinase K for 20 min at 37°C. The slices were washed with PBS and incubated with the working-strength terminal deoxynucleotidyl transferase enzyme at 37°C for 1 h in a humid container. After rinsing the sections in PBS, they were stained with DAPI. Photographs were taken and observations were made using a fluorescence microscope (Olympus). Each experiment was performed using positive and negative controls. An uncolored photo was used as the background reference. Green fluorescence in the ischemic penumbra indicates a positive TUNEL signal. During statistical analysis, the same threshold parameter set was used for all pictures. Three fields from each section were randomly selected for fluorescence intensity determination. The immunofluorescence intensity of TUNEL staining was represented by the mean gray value of the region of interest, which was analyzed using ImageJ software.

### Western blot assay

Approximately 20 mg of cerebral cortex was weighed and transferred into a protein lysate comprising 194 μL radioimmunoprecipitation assay buffer (EpiZyme), 2 μL phenylmethyl sulfonyl fluoride (PMSF, 100 mM, EpiZyme), 2 μL protease inhibitor mix (100 × , EpiZyme), and phosphatase inhibitor mix (100 × , EpiZyme). A standard curve was constructed, protein concentrations were determined using a bicinchoninic acid kit (EpiZyme), and proteins were denatured at 100°C for 10 min after normalizing the concentration of each protein group. Proteins were electrophoresed on 8–12% sodium dodecyl sulfate-polyacrylamide gel electrophoresis, and the gel was transferred to a nitrocellulose (NC) membrane (GE, Germany). The NC membrane was dipped in 5% skim milk for 1 h. After adding primary antibody (GLT-1 dilution 1:2000; phospho-mTOR (Ser2448) (p-mTOR) dilution1:1000; mTOR dilution 1:1000; pAkt dilution 1:1000; Akt dilution 1:1000; pS6 dilution 1:1000; S6 dilution 1:1000; Bax dilution 1:1000; Bcl-2 dilution 1:1000; β-Actin dilution 1:6000; and β-Tublin dilution 1:5000), the membrane was incubated overnight at 4°C on a shaker. The next day, the membranes were incubated for 1 h with HRP-conjugated goat anti-mouse or goat anti-rabbit secondary antibodies. Finally, the blots were visualized using a Bio-Rad ChemiDoc XRS after adding an enhanced chemiluminescent solution (Oriscience, China) to the membrane and analyzed using ImageJ software. GLT-1 protein levels were normalized to β-Actin protein levels, phosphorylated protein levels were normalized to corresponding total protein levels, and Bcl-2 protein levels were normalized to Bax protein levels.

### Enzyme-linked immunosorbent assay (ELISA) analysis

The cerebral cortex was homogenized by adding a medium-strength radioimmunoprecipitation assay buffer cell lysate (FineTest, China) supplemented with a phosphatase inhibitor. The cortex samples were centrifuged at 12000 rpm/min for 5 min at 4°C, and the supernatant was stored at −80°C. Inflammatory cytokine concentrations were evaluated using a commercially supplied ELISA kit (Fine Test), and all experimental procedures were performed following the manufacturer’s instructions [39].

### Statistical analysis

All judgments were blinded. The results are presented as the mean ± standard error of means (SEM). Statistical analysis was conducted using the t-test or one-way analysis of variance, followed by the Tukey test, using GraphPad Prism software (version 9.0). Differences were considered statistically significant at P < 0.05.

## Results

### Inhibition of the mTOR pathway mitigated neurological deficits and cerebral infarct volume in MCAO/R rats

**Fig 3:**
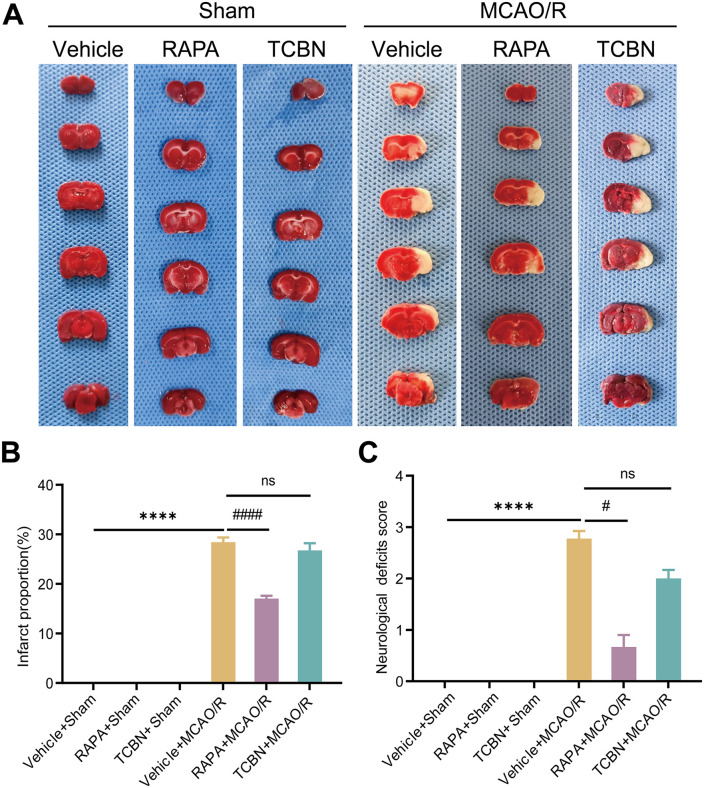
Inhibition of the mTOR pathway conferred neuroprotective effects against cerebral I/R injury. **(A)** Representative image of TTC staining, white color indicates infarct area. **(B)** Statistical plot of infarct volume ratio for TTC staining. **(C)** Longa scores and quantitative statistical plots. n = 3. All plots are presented as the mean ± SEM. ^****^ p < 0.0001 vs. Vehicle + Sham group, ^#^ p < 0.05 and ^####^ p < 0.0001 vs. Vehicle + MCAO/R group, ns: no significant.

Figs 3A and 3B show the images and statistical plots of TTC staining. The normal brain tissue is stained red and infarcted areas are stained white. No significant infarction was identified in the Vehicle + Sham, RAPA + Sham, and TCBN + Sham groups. The infarct volume in the Vehicle + MCAO/R group was 28.43% ± 3.54% of the total brain volume, and no significant change in infarct volume was observed in the TCBN + MCAO/R group (26.76% ± 3.55%) compared with that in the Vehicle + MCAO/R group, whereas the infarct volume decreased by 11.40% ± 3.55% after RAPA pretreatment (p < 0.0001). The Longa scores are shown in Fig 3C. The Vehicle + Sham, RAPA + Sham, and TCBN + Sham groups showed no significant neurological deficits. Longa scores were significantly higher in the Vehicle + MCAO/R group than in the Vehicle + Sham group (p < 0.0001). RAPA treatment substantially reduced neurological scores in MCAO/R rats (p < 0.05). Conversely, neurological function was not significantly enhanced in MCAO/R rats using TCBN treatment. These findings indicate that inhibiting the mTOR pathway may protect against brain I/R injury in rats.

### Inhibition of the mTOR pathway attenuated neuroinflammation in MCAO/R rats

**Fig 4:**
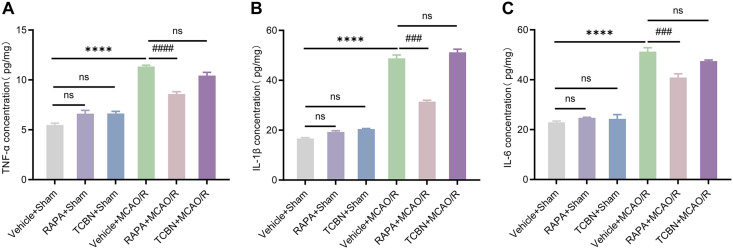
Detection of cortical inflammatory factor levels by ELISA assay. **(A)** Quantitative analysis of TNF-α levels in the cortex. **(B)** Quantitative analysis of IL-1β levels in the cortex. **(C)** Quantitative analysis of IL-6 levels in the cortex. n = 3. All plots are presented as the mean ± SEM. ^****^ p < 0.0001 vs. Vehicle + Sham group, ^###^ p < 0.001 and ^####^ p < 0.0001 vs. Vehicle + MCAO/R group, ns: no significant, ELISA: enzyme-linked immunosorbent assay.

Neuroinflammation is a pivotal mechanism involved in the pathogenesis of I/R injury in the brain. Following cerebral ischemia, activated astrocytes release numerous pro-inflammatory factors, including TNF-α, IL-1β, and IL-6, which exacerbate brain damage [40]. In this study, the levels of three pro-inflammatory factors, TNF-α, IL-1β, and IL-6, were quantified via ELISA in cortical homogenates (Fig 4). The results showed no statistically significant differences in the levels of inflammatory factors among the RAPA + Sham, TCBN + Sham, and Vehicle + Sham groups. A significant increase in inflammatory factor levels was observed after MCAO/R (p < 0.0001). Pretreatment with RAPA reversed the increase in inflammatory factors in the model group (p < 0.001), whereas TCBN pretreatment was not effective in significantly reducing pro-inflammatory factor levels in cerebral ischemic rats. These findings indicate that RAPA decreases the production and release of cortical pro-inflammatory factors after brain I/R injury by inhibiting the mTOR pathway.

### The effects of I/R injury on astrocytes and neuron growth and apoptotic protein

**Fig 5:**
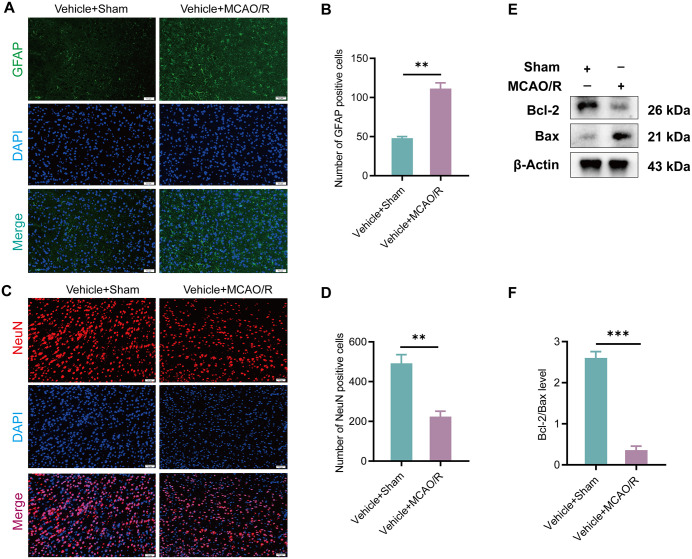
The effects of MCAO/R on astrocytes, neurons, and apoptotic proteins. **(A)** Immunofluorescence staining of GFAP) (green). **(B)** Quantitative analysis of GFAP positive cells. **(C)** Immunofluorescence staining of NeuN) (red). **(D)** Quantitative analysis of NeuN positive cells. Nuclei are labeled with blue fluorescence by DAPI. Scale bar = 50 µm. **(E)** Western blot of Bcl-2 and Bax in ischemic penumbra, **(F)** with subsequent quantitative analysis of Bcl-2/Bax. n = 3. All plots are presented as the mean ± SEM. ^**^ p < 0.01 and ^***^ p < 0.001 vs. Vehicle + Sham group, ns: no significant. GFAP: Glial fibrillary acidic protein; NeuN: neuron-specific nuclear protein; DAPI: 4′,6-Diamidino-2-phenylindole.

We investigated changes in astrocytes, neurons, and apoptotic proteins in the ischemic penumbra after brain I/R. Astrocytes proliferated with significant hypertrophy and increased protrusions following MCAO/R (p < 0.01), whereas the number of neurons was significantly reduced (p < 0.01). Western blot analysis revealed a reduction in the anti-apoptotic protein Bcl-2, an increase in the pro-apoptotic protein Bax, and a notable decrease in the Bcl-2/Bax ratio (P < 0.001). These results indicate that brain I/R injury promotes astrocyte activation and apoptosis, which may contribute to neuronal loss (Fig 5).

### MCAO/R activated the mTOR/Akt signaling pathway in ischemic penumbra and inhibits GLT-1 protein expression

**Fig 6:**
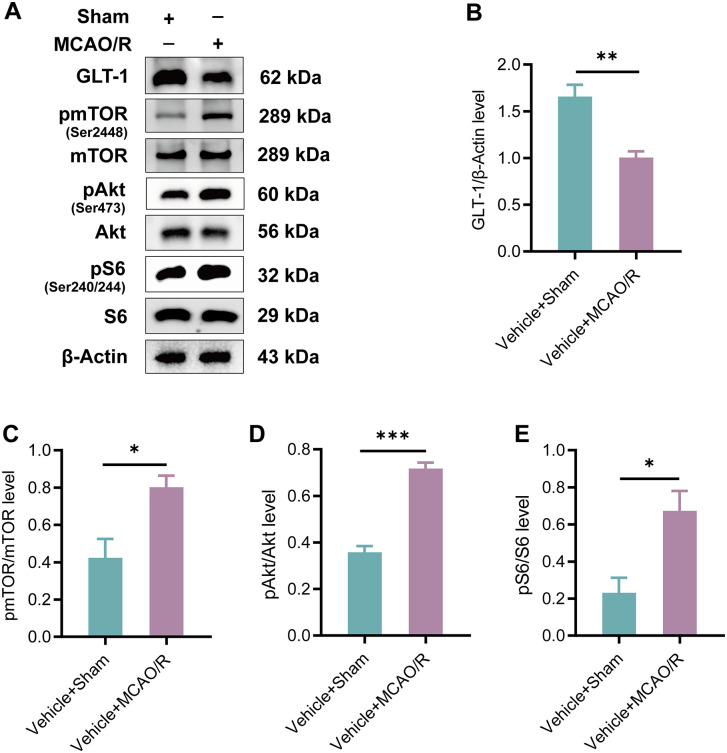
Expression of post-MCAO/R pathway and apoptotic proteins. **(A)** Western blot analysis of GLT-1、p-mTOR、mTOR、p-Akt、Akt、pS6 and S6 in ischemic penumbra. **(B-E)** Quantitative analysis of GLT-1/β-Actin, p-mTOR/mTOR, p-Akt/Akt, and pS6/S6. n = 3. Each lane represents an individual experimental animal. All plots are presented as the mean ± SEM. ^*^ p < 0.05, ^**^ p < 0.01, and ^***^ p < 0.001 vs. Vehicle + Sham group, ns: no significant. MCAO/R: middle cerebral artery occlusion and reperfusion.

We further examined alterations in the mTOR/Akt pathway and GLT-1 proteins in the ischemic penumbra after brain I/R. The expression of p-Akt, p-mTOR, and p-S6 was increased in the MCAO/R group compared with that in the sham group (p < 0.05, Fig 6), while GLT-1 protein expression was significantly reduced (p < 0.01). These findings reveal that brain I/R injury activates the mTOR/Akt signaling pathway in the ischemic penumbra and inhibits GLT-1 protein expression.

### The mTOR/Akt signaling pathway may be potentially involved in regulating astrocyte and neuronal growth under brain I/R conditions

**Fig 7:**
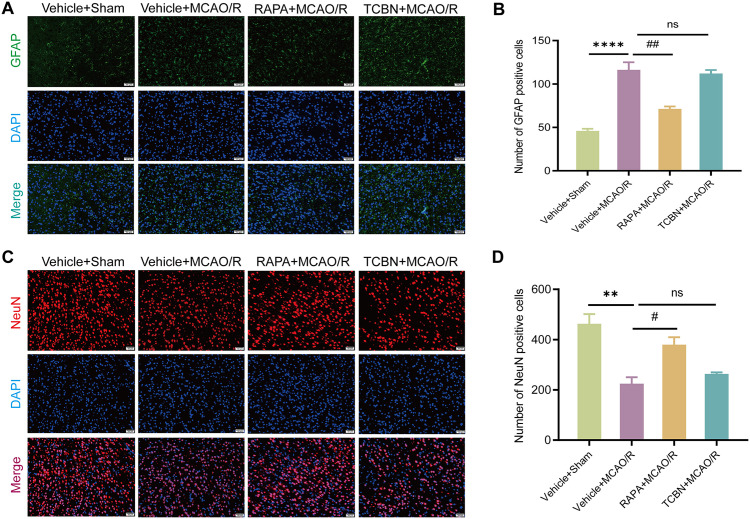
Immunofluorescence staining of astrocytes and neurons in cerebral ischemic rats after pretreatment with RAPA or TCBN. **(A)** Immunofluorescence staining of GFAP (green). **(B)** Quantitative analysis of GFAP-positive cells. **(C)** Immunofluorescence staining of NeuN (red). **(D)** Quantitative analysis of NeuN positive cells. Nuclei are labeled with blue fluorescence by DAPI. Scale bar = 50 µm. n = 3. All plots are presented as the mean ± SEM. ^**^ p < 0.01 and ^****^ p < 0.0001 vs. Vehicle + Sham group, ^#^ p < 0.05 and ^##^ p < 0.01 vs. Vehicle + MCAO/R group, ns: no significant, GFAP: Glial fibrillary acidic protein, NeuN: neuron-specific nuclear protein, DAPI: 4′,6-Diamidino-2-phenylindole.

Immunofluorescent staining was performed to elucidate the molecular mechanisms underlying the regulation of astrocytes and neurons under brain I/R conditions. Astrocyte protrusions were significantly shorter and fewer in number, and the number of cells was significantly reduced in the RAPA + MCAO/R group compared with that in the Vehicle + MCAO/R group (p < 0.0001). In contrast, the number of neurons was significantly increased (p < 0.05). There was no discernible alteration in the number of astrocytes in the TCBN + MCAO/R group (p > 0.05) and no appreciable increase in the number of surviving neurons was observed. Meanwhile, the number of astrocytic protrusions in the TCBN + MCAO/R group was greater, and the protrusions were more obvious. The mTOR pathway is speculated to be involved in the growth of astrocytes and neurons under brain I/R conditions, and inhibiting mTOR signaling alleviates astrocyte activation and promotes neuronal survival after MCAO/R (Fig 7).

### The mTOR/ Akt signaling pathway may be potentially involved in regulating the GLT-1 expression under brain I/R conditions

**Fig 8:**
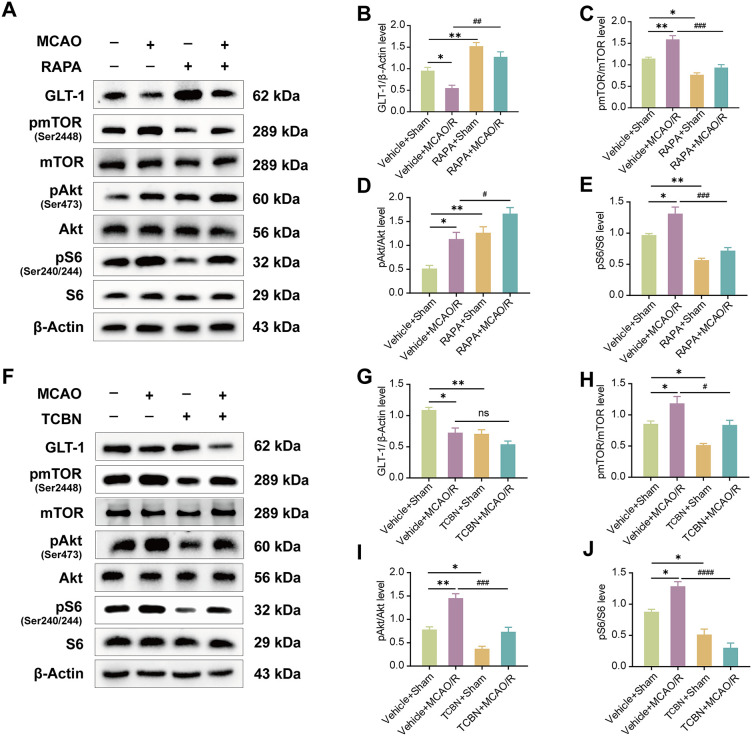
The effects of RAPA or TCBN treatment on GLT-1 expression in MCAO/R rats. **(A)** Western blot analysis of GLT-1、p-mTOR、mTOR、p-Akt、Akt、pS6 and S6 in ischemic penumbra following RAPA treatment. **(B-E)** Quantitative analysis of GLT-1/β-Actin, p-mTOR/mTOR, p-Akt/Akt and pS6/S6 after RAPA treatment. **(F)** Western blot analysis of GLT-1、p-mTOR、mTOR、p-Akt、Akt、pS6, and S6 in ischemic penumbra following TCBN treatment. **(G-J)** Quantitative analysis of GLT-1/β-Actin, p-mTOR/mTOR, p-Akt/Akt and pS6/S6 after TCBN treatment. n = 3. All plots are presented as the mean ± SEM. ^*^ p < 0.05 and ^**^ p < 0.01 vs. Vehicle + Sham group, ^#^ p < 0.05, ^##^ p < 0.01, ^###^ p < 0.001 and ^####^ p < 0.0001 vs. Vehicle + MCAO/R group, ns: no significant.

To determine whether regulating GLT-1 expression during brain I/R injury is related to the mTOR/Akt pathway, we used mTOR inhibitor RAPA and Akt inhibitor TCBN preconditioning to block the pathway prior to MCAO/R. p-S6 was used to determine whether the mTOR pathway was activated. The results showed that p-mTOR, p-Akt, and p-S6 expression increased (p < 0.05) and GLT-1 expression decreased (p < 0.05) in the MCAO/R group. RAPA pretreatment further elevated p-Akt expression (p < 0.05), upregulated GLT-1 expression (p < 0.01), and reduced p-mTOR and p-S6 in the model group (p < 0.001). Nevertheless, TCBN pretreatment inhibited the mTOR/Akt pathway and further reduced the expression of GLT-1 in the model group, resulting in the reversal of the upregulation of p-mTOR, p-Akt, and p-S6 expressions (Fig 8). These results indicate that RAPA activates Akt, which may be necessary for GLT-1 expression. Here, we show the upregulation of GLT-1 expression via the mTOR/Akt signaling cascade. Thus, the mTOR/Akt pathway may be involved in the regulation of GLT-1 expression during brain I/R injury.

### The mTOR/ Akt signaling pathway may be potentially involved in regulating cell apoptosis

**Fig 9:**
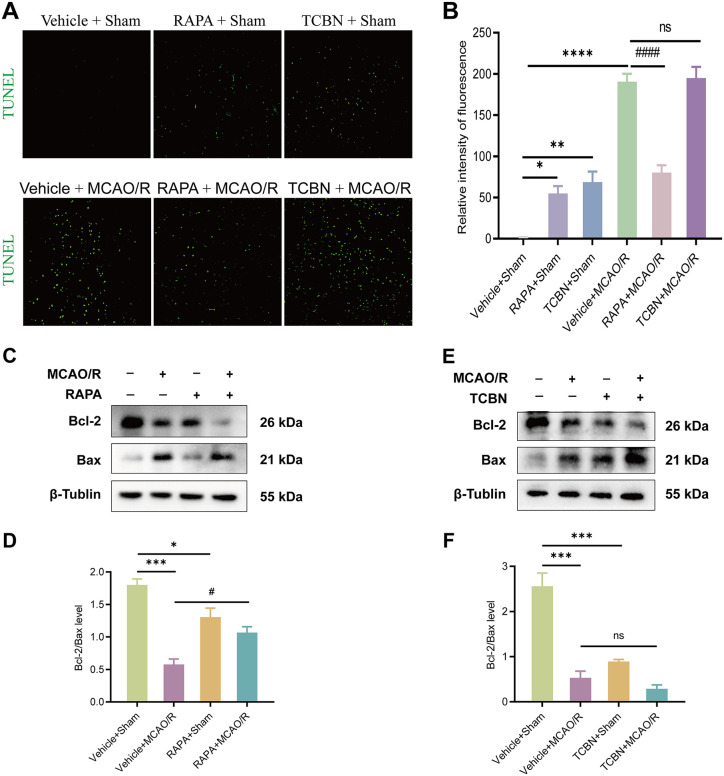
The effect of mTOR/Akt pathway on apoptosis. **(A)** TUNEL immunofluorescent staining (green) of the ischemic penumbra in the cortex after RAPA and TCBN treatment and (B) quantitative analysis of fluorescence intensity. Nuclei are labeled with blue fluorescence by DAPI. **(C)** Western blot analysis of Bcl-2 and Bax in ischemic penumbra following RAPA treatment. **(D)** Quantitative analysis of Bcl-2/Bax after RAPA treatment. **(E)** Western blot analysis of Bcl-2 and Bax in ischemic penumbra following TCBN treatment. **(F)** Quantitative analysis of Bcl-2/Bax after TCBN treatment. n = 3. All plots are presented as the mean ± SEM. ^*^ p < 0.05, ^**^ p < 0.01, ^***^ p < 0.001 and ^****^ p < 0.0001 vs. Vehicle + Sham group, ^#^ p < 0.05 and ^####^ p < 0.0001 vs. Vehicle + MCAO/R group, ns: no significant. DAPI: 4′,6-Diamidino-2-phenylindole, TUNEL: Terminal Deoxynucleotidyl Transferase-mediated dUTP Nick-end Labeling.

The Akt/mTOR pathway is involved in the process of apoptosis [41]. Therefore, we sought to explore the effect of the mTOR/Akt pathway on apoptosis in brain I/R conditions, using TUNEL staining and western blotting. TUNEL fluorescence intensity increased in the RAPA- or TCBN-treated Shan groups relative to that in the Vehicle + Shan group (p < 0.05). Furthermore, the TUNEL fluorescence intensity significantly increased following MCAO/R (p < 0.0001). RAPA pretreatment significantly attenuated TUNEL fluorescence intensity following modeling (p < 0.0001), whereas TCBN pretreatment did not have a statistically significant effect on TUNEL fluorescence intensity in the MCAO/R group. Western blot analysis revealed a significant decrease in the Bcl-2/Bax ratio after MCAO/R (p < 0.001). Inhibition of the mTOR pathway increased the Bcl-2/Bax ratio in ischemic rats (p < 0.05), but Akt pathway inhibition did not protect against apoptosis (p > 0.05) (Fig 9).

### The effects of the mTOR/Akt pathway on astrocytes, neurons, and apoptosis in the sham-operated group

**Fig 10:**
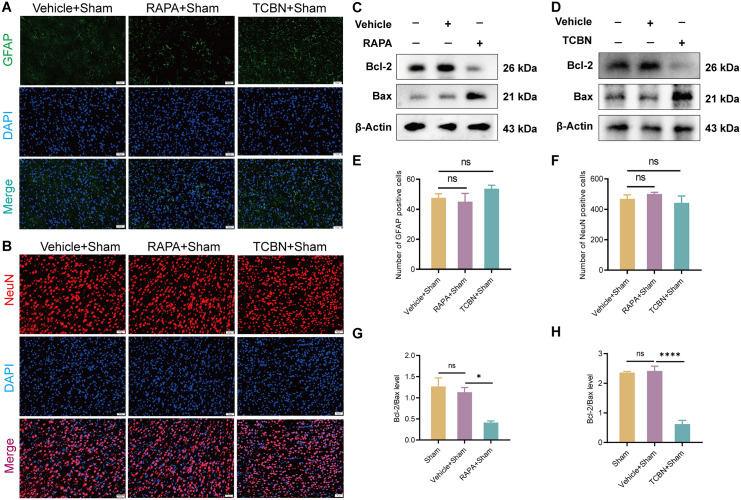
The effects of the mTOR/Akt pathway on astrocytes, neurons, and apoptosis in sham group. **(A)** Immunofluorescence staining of GFAP (green). **(E)** Quantitative analysis of GFAP positive cells. **(B)** Immunofluorescence staining of NeuN) (red). **(F)** Quantitative analysis of NeuN positive cells. Nuclei are labeled with blue fluorescence by DAPI. Scale bar = 50 µm. **(C)** Western blot analysis of Bcl-2 and Bax in the cerebral cortex following rapamycin treatment, **(G)** with subsequent quantitative analysis of Bcl-2/Bax. **(D)** Western blot analysis of Bcl-2 and Bax in the cerebral cortex following triciribine treatment, **(H)** with subsequent quantitative analysis of Bcl-2/Bax. n = 3. All plots are presented as the mean ± SEM. ^*^ p < 0.05 and ^****^ p < 0.0001 vs. Vehicle + Sham group, ns: no significant. GFAP: Glial fibrillary acidic protein; NeuN: neuron-specific nuclear protein; DAPI: 4′,6-Diamidino-2-phenylindole.

To observe the effects of RAPA and TCBN treatment on astrocytes and neurons, we performed immunofluorescence staining for GFAP, expressed by astrocytes, and NeuN, expressed by neurons. No significant difference was observed in the number of GFAP- and NeuN-positive cells between the RAPA- or TCBN-treated sham groups (p > 0.05), and no significant changes were observed in astrocyte processes and neuronal morphology. Western blot data revealed that the Bcl-2/Bax ratio was diminished in the groups treated with RAPA and TCBN compared with that in the control group and that the apoptotic effect of TCBN was particularly notable (p < 0.0001). These results reveal that the mTOR/Akt pathway had no significant effect on astrocytes or neurons in the sham-operated group. Conversely, RAPA and TCBN treatment promoted apoptosis in rats in the sham group (Fig 10).

### The mTOR/Akt pathway plays a role in regulating GLT-1 protein expression in the sham-operated group

**Fig 11:**
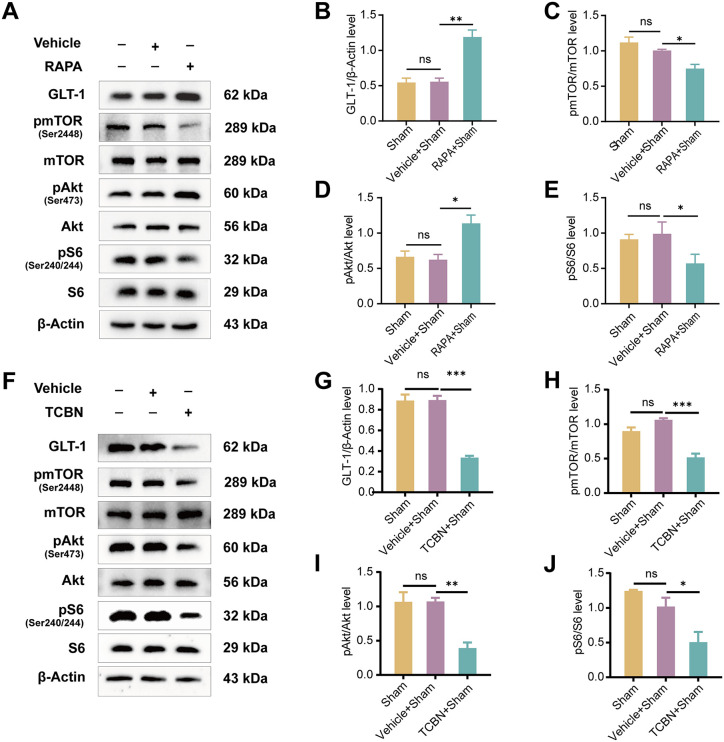
Inhibition of the mTOR pathway up-regulated GLT-1 expression in the sham-operated group. **(A)** Western blot analysis of GLT-1, p-mTOR, mTOR, p-Akt, Akt, pS6 and S6 in the cerebral cortex of the Sham group following RAPA treatment. **(B-E)** Quantitative analysis of GLT-1/β-Actin, p-mTOR/mTOR, p-Akt/Akt, and pS6/S6 after RAPA treatment. **(F)** Western blot analysis of GLT-1, p-mTOR, mTOR, p-Akt, Akt, pS6, and S6 in the cerebral cortex of the Sham group following TCBN treatment. **(G-H)** Quantitative analysis of GLT-1/β-Actin, p-mTOR/mTOR, p-Akt/Akt and pS6/S6 after TCBN treatment. n = 3. Each lane represents an individual experimental animal. All plots are presented as the mean ± SEM. * p < 0.05, ** p < 0.01 and *** p < 0.001 vs. Vehicle + Sham group, ns: no significant.

Subsequently, we plan to investigate the impact of the mTOR/Akt pathway on GLT-1 protein expression. We also focused on the alterations in pathway-related proteins in the rats of the Sham operation group (Fig 11). Detection of p-S6 is a commonly used marker of mTOR pathway activity [42]. Therefore, simultaneous detection of p-S6 protein expression is a valuable approach. The results did not reveal any discernible alterations in GLT-1, p-mTOR, mTOR, pAkt, Akt, pS6, or S6 protein expression in the Vehicle + Sham group compared with that in the sham group. RAPA treatment increased GLT-1 and p-Akt protein expression in the sham group (p < 0.05), whereas p-mTOR and pS6 expression decreased (p < 0.05). Treatment with TCBN, a specific inhibitor of Akt, significantly inhibited the expression of GLT-1, p-mTOR, p-Akt, and p-S6 (P < 0.01). These results indicate that the mTOR/Akt pathway is implicated in regulating GLT-1 expression and that RAPA may enhance the expression level of GLT-1 by activating the Akt pathway in rats belonging to the sham group.

## Discussion

IS is a significant public health concern, with high incidence, disability, and mortality rates worldwide [2]. It poses a serious threat to human life and health. Astrocytes are the most prevalent type of glial cells in the brain and are essential for regulating neuronal survival, cytoplasmic Glu concentration, and inflammatory mediators [14,43]. Excitotoxicity, defined as the excessive Glu accumulation within the extracellular space, is a major mechanism underlying various neurological disorders, including stroke, traumatic brain injury, and epilepsy [44]. GLT-1 is crucial for the uptake of extracellular Glu and the protection of neurons from ischemic injury, primarily within astrocytes. However, the mechanisms governing GLT-1 expression are not well understood. Therefore, the aim of this study was to investigate the interactions and underlying mechanisms between the mTOR/Akt pathway and GLT-1 expression in cases of brain I/R injury, along with their impact on astrocyte growth, with the goal of offering new perspectives for treating ischemic stroke.

In this study, the MCAO/R model was constructed using the Zea Longa method, with the external carotid artery serving as the inlet, to simulate ischemia-reperfusion injury *in vivo*. Using this method, craniotomy can be avoided, reperfusion becomes stable and controllable, and CBF can be monitored using a laser speckle blood flow imaging system before and after modeling to further ensure successful reperfusion. Concurrently, the Longa score was employed to evaluate neurological deficits in rats following modeling to identify rats with successful modeling for subsequent molecular biology and morphology studies. This approach improves the reliability of the experiments. Following cerebral ischemia, the brain cells in the ischemic core undergo irreversible death. In contrast, the brain tissue in the ischemic penumbra is biochemically and metabolically compromised, with a risk of further damage and/or necrosis [14]. However, this tissue is also a target for IS treatment as it may be amenable to recovery. Accordingly, in the present study, we selected the ischemic penumbra as the target region to investigate the mechanisms underlying brain I/R injury and GLT-1 expression, which is more clinically relevant.

GLT-1, a transmembrane transporter protein located in the astrocyte membrane, is responsible for 90% of Glu uptake by astrocytes [45]. It helps maintain Glu in the extracellular space at normal levels and limits its excitotoxicity in the CNS. However, under ischemic hypoxia, adenylate cyclase inhibition and the activation of Glu metabotropic receptors mediate the downregulation of GLT-1 expression in astrocytes, resulting in insufficient clearance of excess Glu from the synaptic gap [46,47]. This exacerbates the excitotoxicity, leading to neuronal death. Recently, a growing body of evidence reveals that the downregulation of GLT-1 expression is closely associated with various neurological disorders and that modulation of GLT-1 expression may assist in alleviating neurological deficits and play a pivotal role in neuroprotection. Liang et al. observed that the Glu uptake activity was diminished in astrocyte cultures derived from the parietal cortex of patients with Alzheimer’s [48]. Furthermore, estrogen treatment delays the onset and progression of Alzheimer’s disease by enhancing Glu uptake by astrocytes through augmented GLT-1 expression [48]. Peterson et al. found that the upregulation of the GLT-1 expression resulted in a reduction in seizure frequency and total seizure time and provided neuroprotective effects at an early stage of epilepsy development [49]. Aizawa et al. discovered that knockdown of GLT-1 in mice resulted in increased sensitivity, frequency, and rate of cortical dilatory inhibition, which underlies the pathogenesis of migraine [50]. Consequently, a reduction in GLT-1 levels may precipitate an increase in the frequency of migraine attacks. GLT-1 expression was also downregulated in rats with stroke in the MCAO/R model, and an increase in GLT-1 expression was demonstrated to reduce ischemic brain injury in these rats [37]. Given the pivotal roles of GLT-1 and astrocytes in neurological disorders, we aimed to assess GLT-1 protein expression in the penumbra of rats with cerebral ischemia. This was achieved using western blotting, whereas immunofluorescence and TUNEL staining were used to analyze astrocyte survival and neuronal damage. The results of our study showed that following MCAO/R, rats showed reduced GLT-1 expression within the ischemic penumbra, an increase in GFAP protein levels and neuronal apoptosis, the release of pro-inflammatory factors, a reduced number of surviving neurons, and a significant increase in neurologic deficit scores. These findings indicate that astrocyte activation and low GLT-1 expression may contribute to exacerbating neurological damage in patients with stroke. Hypoxia inhibited the expression of GLT-1 at protein and mRNA levels [51]. Furthermore, high levels of Glu in the synaptic gap induce the death of GLT-1-expressing astrocytes [52], which may account for the increased activation of astrocytes observed after ischemic-hypoxic brain injury, whereas a marked decrease in GLT-1 expression was observed.

The AKT/mTOR signaling pathway is crucial in regulating cell survival, differentiation, proliferation, and apoptosis [53,54]. It plays a central role in IS pathophysiology. The phosphoinositol-3-kinase (phosphatidylinositol 3-kinase)/Akt/mTOR pathway is common in apoptosis [55,56]. Akt regulates neuronal survival and apoptosis, and upregulation of Akt phosphorylation activates anti-apoptotic mechanisms in cells, thereby reducing neuronal apoptosis and promoting cell survival [57]. mTOR is a 289 kDa serine-threonine kinase that regulates growth factors and nutrient-mediated cell and organismal growth. It assembles into two complexes, mTORC1 and mTORC2, which are categorized by their associated proteins and responsiveness to RAPA [58]. RAPA functions as a specific inhibitor of mTORC1, whereas mTORC2 is insensitive to it [33].

RAPA exerts a dual effect on apoptosis by increasing the levels of the anti-apoptotic protein Bcl-2 in neurodegenerative diseases [59]. Gao et al. reported that RAPA prevented cardiomyocyte apoptosis in rats with chronic heart failure by inhibiting mTOR signaling [60]. Ding et al. found that it suppresses apoptotic neuronal death following traumatic brain injury via the mTOR-p53-Bax axis [61]. Notably, in our previous study, we found that the mTOR-Akt-NF-κ B cascade was involved in regulating astrocyte GLT-1 in OGD and that upregulating GLT-1 expression required Akt activation and a short-term RAPA treatment upregulated the expression of GLT-1 by inhibiting mTOR negative feedback activation of Akt [30]. The present study revealed aberrant activation of the mTOR pathway (increased expression of p-Akt and p-mTOR), activated proliferation of astrocytes, and a significant increase in the expression of the astrocyte marker, GFAP, in the ischemic penumbra of model rats, consistent with findings from previous studies [62,63]. TUNEL staining revealed a notable elevation in apoptosis, a decline in the expression of Bcl-2/Bax, and a pronounced reduction in the number of neurons following MCAO/R. RAPA pretreatment inhibited mTOR expression; however, it promoted Akt phosphorylation at Ser473. This was accompanied by the upregulation of GLT-1 protein expression and the inhibition of astrocyte activation, significantly reducing cerebral infarct volume and neurological deficit scores in rats in the MCAO/R group. It can be reasonably postulated that RAPA upregulates GLT-1 expression via the mTOR/Akt signaling cascade, thereby mitigating apoptosis triggered by cerebral I/R injury. In contrast, treatment with TCBN reduced p-Akt and p-mTOR protein expression, as well as GLT-1 expression, further increasing the level of apoptosis in the model group of rats. It also reduced the number of neurons but did not significantly improve the volume of cerebral infarcts or neurological deficit scores. These results indicate that inhibiting the mTOR pathway can negatively regulate the expression of p-Akt, upregulate the level of GLT-1, and activate the anti-apoptotic mechanism of cells, thus exerting a protective function in the nervous system. However, we only partially accounted for the morphological changes in astrocytes in this study. The relationship between the mTOR-Akt pathway and the morphological changes in astrocytes required a more extensive investigation.

Moreover, neuroinflammation is a significant pathological process that contributes to neurological impairment in acute IS and may represent a promising therapeutic target for reducing inflammatory damage following brain I/R [64,65]. The Akt/mTOR, toll-like receptor, MAPK, and NF-κB pathways are known to mediate inflammatory response during IS [66]. Cerebral ischemia, inflammation, and oxidative stress induce astrocyte activation, which in turn releases various pro-inflammatory factors (TNF-α, IL-1β, IL-6) [40]. These factors promote astrocyte activation and create a cycle of events that exacerbate stroke-associated brain injury. Various pro-inflammatory mediators contribute to the exacerbation of excitotoxicity by inhibiting the expression of GLT-1 in astrocytes and the subsequent reuptake of Glu [67]. Findings from some studies have shown that TNF-α disrupts the blood-brain barrier and stimulates the induction of other inflammatory mediators, which is positively correlated with the degree of ischemic injury [68]. Furthermore, IL-6 levels are correlated with infarct size and poor clinical outcomes [69]. Reportedly, IL-1β enhances intracellular calcium levels through the activation of N-methyl-D-aspartate receptors, which ultimately leads to neuronal cell death [70]. The cerebral cortex has been shown to release elevated levels of pro-inflammatory mediators, which are accompanied by reduced GLT-1 expression, significantly increased cerebral infarct volume, and neurological deficits following MCAO/R. RAPA exerts its anti-inflammatory and anti-astrocyte activation effects by modulating the mTOR-Akt-NF-κB cascade response in astrocytes and promoting GLT-1 expression [71]. Consequently, an increase in activated astrocytes following cerebral ischemia was observed; nonetheless, the resulting release of proinflammatory mediators may have influenced the downregulation of GLT-1 expression. The Sham group has been previously shown to have baseline levels of inflammatory markers [35,72,73] (Fig 4). We believe that this may be associated with stressors such as environmental factors, surgical incisions, and anesthesia. Normal physiological processes in the body involve certain levels of inflammatory markers. They regulate normal immune responses, cell proliferation, differentiation, and signal transduction. In the present study, pro-inflammatory factors were elevated in the model rats compared with those in the sham group. Upon the inhibition of mTOR, the levels of inflammatory cytokines markedly reduced within the cerebral cortex of the model rats. Conversely, levels of TNF-α, IL-1β, and IL-6 remained elevated following Akt pathway inhibition. The data presented here indicate that RAPA may significantly reduce pro-inflammatory factor levels by stimulating Akt phosphorylation at Ser473, thereby negatively regulating the mTOR-Akt cascade, subsequently upregulating GLT-1 expression, and inhibiting astrocyte activation in rat models. TCBN pretreatment inhibited p-Akt expression; therefore, GLT-1 expression remained lower in the TCBN + MCAO/R group, with a more pronounced inflammatory response. Nevertheless, the precise mechanism by which neuroinflammation affects GLT-1 protein levels in brain I/R remains unclear and requires further investigation.

**Fig 12:**
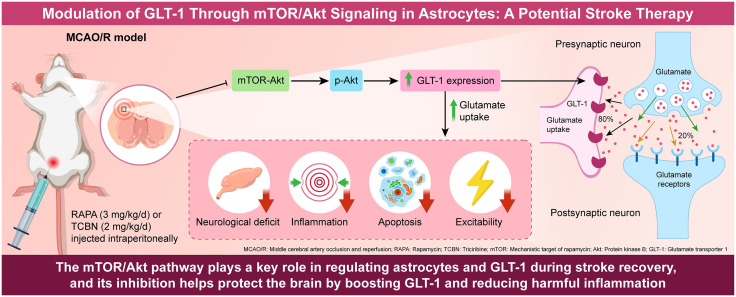
Proposed mechanism of GLT – 1 expression in MCAO rats. **(By Figdraw.)**. Cerebral ischemia initiates the mTOR pathway and encourages the proliferation of astrocytes. By activating the Akt pathway through the mTOR/Akt cascade, Rapamycin promotes the phosphorylation of Akt at the Ser473 site. An increase in GLT-1 expression can be accompanied by a reduction in cerebral ischemia-reperfusion injury.

In conclusion, our results showed that the mTOR-Akt cascade is involved in regulating astrocyte growth and GLT-1 expression under brain I/R conditions. mTOR pathway inhibition can trigger the negative feedback activation of Akt, thereby promoting the expression of GLT-1 and exerting neuroprotective effects (Fig 12). It can be reasonably inferred that upregulating GLT-1 expression serves as a potential target for enhancing IS neurological function. A limitation of the current study is the absence of data on GLT-1 expression under combined rapamycin and triciribine treatment in sham-operated or MCAO/R rats. However, given the established role of the mTOR-Akt-NF-κB cascade in GLT-1 regulation, we predict that triciribine would block the rapamycin-induced upregulation. Future studies are warranted to test this prediction under both physiological and MCAO/R conditions.

## Supporting information

S1 File PLOSOne_Human_Subjects_Research_Checklist. (DOCX)

## References

[1] Cai D, Fraunfelder M, Fujise K, Chen S-Y. ADAR1 exacerbates ischemic brain injury via astrocyte-mediated neuron apoptosis. Redox Biol. 2023;67:102903. doi: 10.1016/j.redox.2023.102903 37801857 PMC10570147

[2] Tsao CW, Aday AW, Almarzooq ZI, Anderson CAM, Arora P, Avery CL, et al. Heart disease and stroke statistics-2023 update: A report from the American Heart Association. Circulation. 2023;147:e93–621. doi: 10.1161/CIR.0000000000001123 36695182 PMC12135016

[3] Amarenco P, Bogousslavsky J, Caplan LR, Donnan GA, Hennerici MG. Classification of stroke subtypes. Cerebrovasc Dis. 2009;27(5):493–501. doi: 10.1159/000210432 19342825

[4] Barthels D, Das H. Current advances in ischemic stroke research and therapies. Biochim Biophys Acta Mol Basis Dis. 2020;1866(4):165260. doi: 10.1016/j.bbadis.2018.09.012 31699365 PMC6981280

[5] GBD 2016 Neurology Collaborators. Global, regional, and national burden of neurological disorders, 1990-2016: A systematic analysis for the Global Burden of Disease Study 2016. Lancet Neurol. 2019;18:459–80. doi: 10.1016/S1474-4422(18)30499-X 30879893 PMC6459001

[6] Ma J, Tian Z, Chai P, Wan Q, Zhai T, Guo F, et al. Estimating the economic burden of stroke in China: a cost-of-illness study. BMJ Open. 2024;14(3):e080634. doi: 10.1136/bmjopen-2023-080634 38485178 PMC10941115

[7] Wu M, Zhang H, Kai J, Zhu F, Dong J, Xu Z, et al. Rapamycin prevents cerebral stroke by modulating apoptosis and autophagy in penumbra in rats. Ann Clin Transl Neurol. 2017;5(2):138–46. doi: 10.1002/acn3.507 29468175 PMC5817831

[8] Fang MC, Cutler DM, Rosen AB. Trends in thrombolytic use for ischemic stroke in the United States. J Hosp Med. 2010;5(7):406–9. doi: 10.1002/jhm.689 20578049 PMC3024589

[9] Campbell BCV, De Silva DA, Macleod MR, Coutts SB, Schwamm LH, Davis SM, et al. Ischaemic stroke. Nat Rev Dis Primers. 2019;5(1):70. doi: 10.1038/s41572-019-0118-8 31601801

[10] Xu D, Kong T, Shao Z, Liu M, Zhang R, Zhang S, et al. Orexin-A alleviates astrocytic apoptosis and inflammation via inhibiting OX1R-mediated NF-κB and MAPK signaling pathways in cerebral ischemia/reperfusion injury. Biochim Biophys Acta Mol Basis Dis. 2021;1867(11):166230. doi: 10.1016/j.bbadis.2021.166230 34358627

[11] Neuhaus AA, Couch Y, Hadley G, Buchan AM. Neuroprotection in stroke: the importance of collaboration and reproducibility. Brain. 2017;140(8):2079–92. doi: 10.1093/brain/awx126 28641383

[12] Hill MD, Goyal M, Menon BK, Nogueira RG, McTaggart RA, Demchuk AM, et al. Efficacy and safety of nerinetide for the treatment of acute ischaemic stroke (ESCAPE-NA1): a multicentre, double-blind, randomised controlled trial. Lancet. 2020;395(10227):878–87. doi: 10.1016/S0140-6736(20)30258-0 32087818

[13] Sofroniew MV, Vinters HV. Astrocytes: biology and pathology. Acta Neuropathol. 2010;119(1):7–35. doi: 10.1007/s00401-009-0619-8 20012068 PMC2799634

[14] Patabendige A, Singh A, Jenkins S, Sen J, Chen R. Astrocyte Activation in Neurovascular Damage and Repair Following Ischaemic Stroke. Int J Mol Sci. 2021;22(8):4280. doi: 10.3390/ijms22084280 33924191 PMC8074612

[15] Poskanzer KE, Molofsky AV. Dynamism of an Astrocyte In Vivo: Perspectives on Identity and Function. Annu Rev Physiol. 2018;80:143–57. doi: 10.1146/annurev-physiol-021317-121125 29166242 PMC5811396

[16] Liu Z, Chopp M. Astrocytes, therapeutic targets for neuroprotection and neurorestoration in ischemic stroke. Prog Neurobiol. 2016;144:103–20. doi: 10.1016/j.pneurobio.2015.09.008 26455456 PMC4826643

[17] Qiu J, Yan Z, Tao K, Li Y, Li Y, Li J, et al. Sinomenine activates astrocytic dopamine D2 receptors and alleviates neuroinflammatory injury via the CRYAB/STAT3 pathway after ischemic stroke in mice. J Neuroinflammation. 2016;13(1):263. doi: 10.1186/s12974-016-0739-8 27724964 PMC5057372

[18] Goldshmit Y, Kanner S, Zacs M, Frisca F, Pinto AR, Currie PD, et al. Rapamycin increases neuronal survival, reduces inflammation and astrocyte proliferation after spinal cord injury. Mol Cell Neurosci. 2015;68:82–91. doi: 10.1016/j.mcn.2015.04.006 25936601

[19] Zhang X, Wei M, Fan J, Yan W, Zha X, Song H, et al. Ischemia-induced upregulation of autophagy preludes dysfunctional lysosomal storage and associated synaptic impairments in neurons. Autophagy. 2021;17(6):1519–42. doi: 10.1080/15548627.2020.1840796 33111641 PMC8205014

[20] Bristot Silvestrin R, Bambini-Junior V, Galland F, Daniele Bobermim L, Quincozes-Santos A, Torres Abib R, et al. Animal model of autism induced by prenatal exposure to valproate: altered glutamate metabolism in the hippocampus. Brain Res. 2013;1495:52–60. doi: 10.1016/j.brainres.2012.11.048 23219577

[21] Mironova YS, Zhukova IA, Zhukova NG, Alifirova VM, Izhboldina OP, Latypova AV. Parkinson’s disease and glutamate excitotoxicity. Zh Nevrol Psikhiatr Im S S Korsakova. 2018;118(6. Vyp. 2):50–4. doi: 10.17116/jnevro201811806250 30346434

[22] Pajarillo E, Rizor A, Lee J, Aschner M, Lee E. The role of astrocytic glutamate transporters GLT-1 and GLAST in neurological disorders: Potential targets for neurotherapeutics. Neuropharmacology. 2019;161:107559. doi: 10.1016/j.neuropharm.2019.03.002 30851309 PMC6731169

[23] Dong X, Wang Y, Qin Z. Molecular mechanisms of excitotoxicity and their relevance to pathogenesis of neurodegenerative diseases. Acta Pharmacol Sin. 2009;30(4):379–87. doi: 10.1038/aps.2009.24 19343058 PMC4002277

[24] Mahmoud S, Gharagozloo M, Simard C, Gris D. Astrocytes Maintain Glutamate Homeostasis in the CNS by Controlling the Balance between Glutamate Uptake and Release. Cells. 2019;8(2):184. doi: 10.3390/cells8020184 30791579 PMC6406900

[25] Rao P, Yallapu MM, Sari Y, Fisher PB, Kumar S. Designing Novel Nanoformulations Targeting Glutamate Transporter Excitatory Amino Acid Transporter 2: Implications in Treating Drug Addiction. J Pers Nanomed. 2015;1(1):3–9. 26635971 PMC4666545

[26] Yu D, Fan Y, Sun X, Yao L, Chai W. Effects of erythropoietin preconditioning on rat cerebral ischemia-reperfusion injury and the GLT-1/GLAST pathway. Exp Ther Med. 2016;11(2):513–8. doi: 10.3892/etm.2015.2919 26893639 PMC4734197

[27] Xian X-H, Gao J-X, Qi J, Fan S-J, Zhang M, Li W-B. Activation of p38 MAPK participates in the sulbactam-induced cerebral ischemic tolerance mediated by glial glutamate transporter-1 upregulation in rats. Sci Rep. 2020;10(1):20601. doi: 10.1038/s41598-020-77583-0 33244020 PMC7692545

[28] Lee E, Sidoryk-Wegrzynowicz M, Yin Z, Webb A, Son D-S, Aschner M. Transforming growth factor-α mediates estrogen-induced upregulation of glutamate transporter GLT-1 in rat primary astrocytes. Glia. 2012;60(7):1024–36. doi: 10.1002/glia.22329 22488924 PMC3353324

[29] Karki P, Hong P, Johnson J, Pajarillo E, Son DS, Aschner M, et al. Arundic acid increases expression and function of astrocytic glutamate transporter EAAT1 via the ERK, Akt, and NF-kappaB pathways. Molecular Neurobiology. 2018;55:5031–46. doi: 10.1007/s12035-017-0709-x 28812276 PMC5964991

[30] Ji Y-F, Zhou L, Xie Y-J, Xu S-M, Zhu J, Teng P, et al. Upregulation of glutamate transporter GLT-1 by mTOR-Akt-NF-кB cascade in astrocytic oxygen-glucose deprivation. Glia. 2013;61(12):1959–75. doi: 10.1002/glia.22566 24108520

[31] Li M, Yu J, Deng H, Xie S, Li Q, Zhao Y, et al. Upregulation of glutamate transporter 1 by mTOR/Akt pathway in astrocyte culture during oxygen-glucose deprivation and reoxygenation. Exp Brain Res. 2023;241(1):201–9. doi: 10.1007/s00221-022-06514-4 36436003

[32] Koellhoffer EC, McCullough LD. The effects of estrogen in ischemic stroke. Transl Stroke Res. 2013;4(4):390–401. doi: 10.1007/s12975-012-0230-5 24323337 PMC4275797

[33] Yang X, Hei C, Liu P, Song Y, Thomas T, Tshimanga S, et al. Inhibition of mTOR Pathway by Rapamycin Reduces Brain Damage in Rats Subjected to Transient Forebrain Ischemia. Int J Biol Sci. 2015;11(12):1424–35. doi: 10.7150/ijbs.12930 26681922 PMC4672000

[34] Li H, Min J, Mao X, Wang X, Yang Y, Chen Y. Edaravone ameliorates experimental autoimmune thyroiditis in rats through HO-1-dependent STAT3/PI3K/Akt pathway. Am J Transl Res. 2018;10(7):2037–46. 30093941 PMC6079139

[35] Yu L, Zhang Y, Chen Q, He Y, Zhou H, Wan H, et al. Formononetin protects against inflammation associated with cerebral ischemia-reperfusion injury in rats by targeting the JAK2/STAT3 signaling pathway. Biomed Pharmacother. 2022;149:112836. doi: 10.1016/j.biopha.2022.112836 35339827

[36] Li X, Luo P, Wang F, Yang Q, Li Y, Zhao M, et al. Inhibition of N-myc downstream-regulated gene-2 is involved in an astrocyte-specific neuroprotection induced by sevoflurane preconditioning. Anesthesiology. 2014;121(3):549–62. doi: 10.1097/ALN.0000000000000314 24866406

[37] Peng M, Ling X, Song R, Gao X, Liang Z, Fang F, et al. Upregulation of GLT-1 via PI3K/Akt Pathway Contributes to Neuroprotection Induced by Dexmedetomidine. Front Neurol. 2019;10:1041. doi: 10.3389/fneur.2019.01041 31611842 PMC6776610

[38] Longa EZ, Weinstein PR, Carlson S, Cummins R. Reversible middle cerebral artery occlusion without craniectomy in rats. Stroke. 1989;20(1):84–91. doi: 10.1161/01.str.20.1.84 2643202

[39] Dash UC, Swain SK, Jena AB, Dandapat J, Sahoo AK. The ameliorative effect of Piper trioicum in attenuating cognitive deficit in scopolamine induced neurotoxicity in experimental rats. J Ethnopharmacol. 2024;318(Pt A):116911. doi: 10.1016/j.jep.2023.116911 37451488

[40] Rossi D. Astrocyte physiopathology: At the crossroads of intercellular networking, inflammation and cell death. Prog Neurobiol. 2015;130:86–120. doi: 10.1016/j.pneurobio.2015.04.003 25930681

[41] Zhang Y, Cai W, Han G, Zhou S, Li J, Chen M, et al. Panax notoginseng saponins prevent senescence and inhibit apoptosis by regulating the PI3K‑AKT‑mTOR pathway in osteoarthritic chondrocytes. Int J Mol Med. 2020;45(4):1225–36. doi: 10.3892/ijmm.2020.4491 32124939

[42] Marosvári D, Nagy N, Kriston C, Deák B, Hajdu M, Bödör C, et al. Discrepancy Between Low Levels of mTOR Activity and High Levels of P-S6 in Primary Central Nervous System Lymphoma May Be Explained by PAS Domain-Containing Serine/Threonine-Protein Kinase-Mediated Phosphorylation. J Neuropathol Exp Neurol. 2018;77(4):268–73. doi: 10.1093/jnen/nlx121 29361117

[43] Assefa BT, Gebre AK, Altaye BM. Reactive Astrocytes as Drug Target in Alzheimer’s Disease. Biomed Res Int. 2018;2018:4160247. doi: 10.1155/2018/4160247 29888263 PMC5977027

[44] Krzyżanowska W, Pomierny B, Bystrowska B, Pomierny-Chamioło L, Filip M, Budziszewska B, et al. Ceftriaxone- and N-acetylcysteine-induced brain tolerance to ischemia: Influence on glutamate levels in focal cerebral ischemia. PLoS One. 2017;12(10):e0186243. doi: 10.1371/journal.pone.0186243 29045497 PMC5646803

[45] Tejeda-Bayron FA, Rivera-Aponte DE, Malpica-Nieves CJ, Maldonado-Martínez G, Maldonado HM, Skatchkov SN, et al. Activation of Glutamate Transporter-1 (GLT-1) Confers Sex-Dependent Neuroprotection in Brain Ischemia. Brain Sci. 2021;11(1):76. doi: 10.3390/brainsci11010076 33429955 PMC7827447

[46] Taheri F, Sattari E, Hormozi M, Ahmadvand H, Bigdeli MR, Kordestani-Moghadam P, et al. Dose-Dependent Effects of Astaxanthin on Ischemia/Reperfusion Induced Brain Injury in MCAO Model Rat. Neurochem Res. 2022;47(6):1736–50. doi: 10.1007/s11064-022-03565-5 35286515

[47] Wang Y, Lu S, Chen Y, Li L, Li X, Qu Z, et al. Smoothened is a therapeutic target for reducing glutamate toxicity in ischemic stroke. Sci Transl Med. 2021;13(610):eaba3444. doi: 10.1126/scitranslmed.aba3444 34516830

[48] Liang Z, Valla J, Sefidvash-Hockley S, Rogers J, Li R. Effects of estrogen treatment on glutamate uptake in cultured human astrocytes derived from cortex of Alzheimer’s disease patients. J Neurochem. 2002;80(5):807–14. doi: 10.1046/j.0022-3042.2002.00779.x 11948244

[49] Peterson AR, Binder DK. Regulation of Synaptosomal GLT-1 and GLAST during Epileptogenesis. Neuroscience. 2019;411:185–201. doi: 10.1016/j.neuroscience.2019.05.048 31158434

[50] Aizawa H, Sun W, Sugiyama K, Itou Y, Aida T, Cui W, et al. Glial glutamate transporter GLT-1 determines susceptibility to spreading depression in the mouse cerebral cortex. Glia. 2020;68(12):2631–42. doi: 10.1002/glia.23874 32585762

[51] Chao X, Fei F, Fei Z. The role of excitatory amino acid transporters in cerebral ischemia. Neurochem Res. 2010;35(8):1224–30. doi: 10.1007/s11064-010-0178-3 20440555

[52] Krzyzanowska W, Pomierny B, Budziszewska B, Filip M, Pera J. N-Acetylcysteine and Ceftriaxone as Preconditioning Strategies in Focal Brain Ischemia: Influence on Glutamate Transporters Expression. Neurotox Res. 2016;29(4):539–50. doi: 10.1007/s12640-016-9602-z 26861954 PMC4820483

[53] Wu Y-J, Wong B-S, Yea S-H, Lu C-I, Weng S-H. Sinularin Induces Apoptosis through Mitochondria Dysfunction and Inactivation of the pI3K/Akt/mTOR Pathway in Gastric Carcinoma Cells. Mar Drugs. 2016;14(8):142. doi: 10.3390/md14080142 27472346 PMC4999903

[54] Yan Y-T, Li S-D, Li C, Xiong Y-X, Lu X-H, Zhou X-F, et al. Panax notoginsenoside saponins Rb1 regulates the expressions of Akt/ mTOR/PTEN signals in the hippocampus after focal cerebral ischemia in rats. Behav Brain Res. 2018;345:83–92. doi: 10.1016/j.bbr.2018.02.037 29501622

[55] Shao Z-Q, Dou S-S, Zhu J-G, Wang H-Q, Wang C-M, Cheng B-H, et al. Apelin-13 inhibits apoptosis and excessive autophagy in cerebral ischemia/reperfusion injury. Neural Regen Res. 2021;16(6):1044–51. doi: 10.4103/1673-5374.300725 33269749 PMC8224111

[56] Wang Y, Zhao M, Shang L, Zhang Y, Huang C, He Z, et al. Homer1a protects against neuronal injury via PI3K/AKT/mTOR signaling pathway. Int J Neurosci. 2020;130(6):621–30. doi: 10.1080/00207454.2019.1702535 32013638

[57] Sinha D, Kalimutho M, Bowles J, Chan A-L, Merriner DJ, Bain AL, et al. Cep55 overexpression causes male-specific sterility in mice by suppressing Foxo1 nuclear retention through sustained activation of PI3K/Akt signaling. FASEB J. 2018;32(9):4984–99. doi: 10.1096/fj.201701096RR 29683733

[58] Villa-González M, Martín-López G, Pérez-Álvarez MJ. Dysregulation of mTOR Signaling after Brain Ischemia. Int J Mol Sci. 2022;23(5):2814. doi: 10.3390/ijms23052814 35269956 PMC8911477

[59] Chong ZZ, Shang YC, Wang S, Maiese K. Shedding new light on neurodegenerative diseases through the mammalian target of rapamycin. Prog Neurobiol. 2012;99(2):128–48. doi: 10.1016/j.pneurobio.2012.08.001 22980037 PMC3479314

[60] Gao G, Chen W, Yan M, Liu J, Luo H, Wang C, et al. Rapamycin regulates the balance between cardiomyocyte apoptosis and autophagy in chronic heart failure by inhibiting mTOR signaling. Int J Mol Med. 2020;45: 195–209. doi: 10.3892/ijmm.2019.4407 31746373 PMC6889932

[61] Ding K, Wang H, Wu Y, Zhang L, Xu J, Li T, et al. Rapamycin protects against apoptotic neuronal death and improves neurologic function after traumatic brain injury in mice via modulation of the mTOR-p53-Bax axis. J Surg Res. 2015;194(1):239–47. doi: 10.1016/j.jss.2014.09.026 25438952

[62] Li J, Xu P, Hong Y, Xie Y, Peng M, Sun R, et al. Lipocalin-2-mediated astrocyte pyroptosis promotes neuroinflammatory injury via NLRP3 inflammasome activation in cerebral ischemia/reperfusion injury. J Neuroinflammation. 2023;20(1):148. doi: 10.1186/s12974-023-02819-5 37353794 PMC10288712

[63] Zhang Y, Li D, Gao H, Zhao H, Zhang S, Li T. Rapamycin Alleviates Neuronal Injury and Modulates Microglial Activation After Cerebral Ischemia. Mol Neurobiol. 2024;61(8):5699–717. doi: 10.1007/s12035-023-03904-9 38224443

[64] Chamorro Á, Dirnagl U, Urra X, Planas AM. Neuroprotection in acute stroke: targeting excitotoxicity, oxidative and nitrosative stress, and inflammation. Lancet Neurol. 2016;15(8):869–81. doi: 10.1016/S1474-4422(16)00114-9 27180033

[65] Sun Y-Y, Zhu H-J, Zhao R-Y, Zhou S-Y, Wang M-Q, Yang Y, et al. Remote ischemic conditioning attenuates oxidative stress and inflammation via the Nrf2/HO-1 pathway in MCAO mice. Redox Biol. 2023;66:102852. doi: 10.1016/j.redox.2023.102852 37598463 PMC10462885

[66] Mo Y, Sun Y-Y, Liu K-Y. Autophagy and inflammation in ischemic stroke. Neural Regen Res. 2020;15(8):1388–96. doi: 10.4103/1673-5374.274331 31997797 PMC7059569

[67] Wang Z, Pekarskaya O, Bencheikh M, Chao W, Gelbard HA, Ghorpade A, et al. Reduced expression of glutamate transporter EAAT2 and impaired glutamate transport in human primary astrocytes exposed to HIV-1 or gp120. Virology. 2003;312(1):60–73. doi: 10.1016/s0042-6822(03)00181-8 12890621

[68] Zaremba J, Skrobanski P, Losy J. Tumour necrosis factor-alpha is increased in the cerebrospinal fluid and serum of ischaemic stroke patients and correlates with the volume of evolving brain infarct. Biomed Pharmacother. 2001;55(5):258–63. doi: 10.1016/s0753-3322(01)00058-0 11428551

[69] Huang J, Upadhyay UM, Tamargo RJ. Inflammation in stroke and focal cerebral ischemia. Surg Neurol. 2006;66(3):232–45. doi: 10.1016/j.surneu.2005.12.028 16935624

[70] Viviani B, Bartesaghi S, Gardoni F, Vezzani A, Behrens MM, Bartfai T, et al. Interleukin-1beta enhances NMDA receptor-mediated intracellular calcium increase through activation of the Src family of kinases. J Neurosci. 2003;23(25):8692–700. doi: 10.1523/JNEUROSCI.23-25-08692.2003 14507968 PMC6740426

[71] Zhang Y, He X, Wu X, Lei M, Wei Z, Zhang X, et al. Rapamycin upregulates glutamate transporter and IL-6 expression in astrocytes in a mouse model of Parkinson’s disease. Cell Death Dis. 2017;8(2):e2611. doi: 10.1038/cddis.2016.491 28182002 PMC5386462

[72] Singh D. Astrocytic and microglial cells as the modulators of neuroinflammation in Alzheimer’s disease. J Neuroinflammation. 2022;19(1):206. doi: 10.1186/s12974-022-02565-0 35978311 PMC9382837

[73] Li L, Sun L, Qiu Y, Zhu W, Hu K, Mao J. Protective Effect of Stachydrine Against Cerebral Ischemia-Reperfusion Injury by Reducing Inflammation and Apoptosis Through P65 and JAK2/STAT3 Signaling Pathway. Front Pharmacol. 2020;11:64. doi: 10.3389/fphar.2020.00064 32132924 PMC7041339

